# Wound Healing Complications After Sternotomy—Causes, Prevention, and Treatment—A New Look at an Old Problem

**DOI:** 10.3390/jcm13237431

**Published:** 2024-12-06

**Authors:** Agata Jęczmyk, Sebastian Krych, Małgorzata Jekiełek, Michał Jurkiewicz, Paweł Kowalczyk, Karol Kramkowski, Tomasz Hrapkowicz

**Affiliations:** 1 Students’ Scientific Association, III Department of Cardiology, School of Medical Sciences in Zabrze, Medical University of Silesia, 40-055 Katowice, Poland; aga.jeczmyk@gmail.com; 2 Student’s Scientific Association, Department of Cardiac, Vascular and Endovascular Surgery and Transplantology, Faculty of Medical Sciences in Zabrze, Medical University of Silesia, 40-055 Katowice, Poland; jurkiewicz.m@yahoo.com; 3 Silesian Centre for Heart Diseases in Zabrze, Department of Cardiac, Vascular and Endovascular Surgery and Transplantology, Medical University of Silesia, 40-055 Katowice, Poland; thrapkowicz@sum.edu.pl; 4 Department of Physiotherapy, Faculty of Health Sciences, Jagiellonian University Collegium Medicum, 31-008 Cracow, Poland; malgorzata.jekielek@gmail.com; 5 Department of Animal Nutrition, The Kielanowski Institute of Animal Physiology and Nutrition, Polish Academy of Sciences, Instytucka 3, 05-110 Jabłonna, Poland; p.kowalczyk@ifzz.pl; 6 Department of Physical Chemistry, Medical University of Bialystok, Kilińskiego 1, 15-089 Białystok, Poland; kkramk@wp.pl

**Keywords:** thoracic surgery, sternotomy, wound infection, coronary artery bypass, treatment outcome

## Abstract

Median sternotomy is one of the most common procedures in cardiac surgery. This corresponds to the relatively high frequency of infections where surgical incisions are performed. In the prevention of healing disorders, the medical staff intervention is important, as is the patient. The management of wound infection after sternotomy requires a holistic approach. It requires the implementation of adequate antibiotic therapy, surgical treatment of the wound, and, if necessary, reconstruction of tissue defects using skin, muscle, and skin–muscle grafts or greater omentum. The prevention of surgical site infection should be based on asepsis and antisepsis at every stage of surgical treatment; the elimination of modifiable risk factors; and an appropriate, staged, and tension-free technique of chest closure. The described actions are aimed at avoiding the most serious complication associated with a high mortality rate in the form of mediastinitis. The therapeutic procedures are strictly dependent on the degree of tissue involvement and the presented clinical manifestation. During the preparation of this manuscript, scientific publications available on the Pubmed platform were analyzed. The scope of the search was limited to the years 2014–2024. The key words were “sternotomy wound infection”. A total of 114 publications were analyzed, and 56 of them were included. A total of 23 papers were used to discuss the topic.

## 1. Introduction

The mediastinum is an area bounded by the pleura, sternum, spine, superior thoracic aperture, and diaphragm. It is divided into anterior and posterior mediastinal cavities. The anterior contains the heart with the pericardium, the superior and inferior vena cava, the ascending aorta, pulmonary vessels, and phrenic nerves. The posterior contains the descending aorta, esophagus, trachea, vagus, and visceral nerves. The sternum is a flat bone located in the middle of the chest. All bone structures are covered by several layers of muscle [1]. Surgical access via median sternotomy requires cutting skin, subcutaneous tissue, muscles, and the sternum. The right opening and closing technique was dependent on the surgeon, influencing the subsequent wound healing process. Most risk factors were related to patients’ lifestyle and postoperative clinical investigation. Appropriate early-risk-factor identification is crucial to minimizing wound complications. In the case of diagnosing a sternal wound infection, it is necessary to implement adequate treatment as soon as possible in order to avoid a life-threatening complication, mediastinitis. The analyses in the publications considered strictly the topics of risk factors, the clinical picture, or the treatment method of wound infection after median sternotomy. During the preparation of the manuscript, an attempt was made to synthesize the available information into a single source. This is intended to make it easier for clinicians to access the most important data on the etiology, prevention, and treatment of sternotomy wound infections.

## 2. Material and Methods

### 2.1. Search Strategy

During the preparation of this manuscript, scientific publications available on the Pubmed platform were analyzed. The scope of the search was limited to the years 2014–2024. The key words used for the research were “sternotomy”, “wound”, and “infection”.

### 2.2. Study Selection

The selection process analyzed both the abstracts and the main text of each article.

Inclusion criteria were clinical trials; meta-analysis; randomized controlled trials; reviews; and systematic reviews describing etiology, pathogenesis, and therapeutic treatment in wound infection after median sternotomy in coronary artery bypass grafting surgery.

Articles that analyzed other cardiac surgery access, types of surgery (for example, acute aortic aneurysm surgery), and infection with primary skin disease and non-statistical and low statistical power analyses due to lack of data were excluded.

### 2.3. Data Extraction

Selected articles were included in the manuscript taking into consideration identification data, compliance with keywords, statistically significant analyses, and adequately large study groups. A total of 114 publications were analyzed, and 56 of them were included. A total of 23 papers were used to discuss the topic. The paper by Gorlitzer M et al. [2] from 2013 was older than accepted scope of the search and was included due to it being a large prospective randomized multicenter trial.

Two references were included without regard to the inclusion/exclusion criteria. First was book, describing anatomy of mediastinum, and second was CDC/NHSN Surveillance Definitions for Specific Types of Infections supplements about international mediastinitis diagnosis criteria.

References [3,4,5,6,7,8,9,10,11,12,13,14,15,16,17,18,19] were added outside of the accepted search criteria to discuss wound healing processes in detail.

## 3. Median Sternotomy: Application and Access Technique

Median sternotomy is one of the most common incision techniques in cardiac surgery. The procedure begins with the first incision in the previously determined midline along the entire length of the sternum, cutting the epidermis and dermis. The exposure of the sternum requires the resection of adipose tissue using electrocautery. The prepared bone is cut with an alternating saw, and the edges of the sternum are usually covered with surgical wax to provide initial hemostatic protection of the surgical field. The edges of the cut sternum are widened using a retractor. Finally, the fat body or thymus and the pericardial sac are cut [20].

## 4. Pathophysiology of Wound Healing After Sternotomy

Wound healing is a complex biological process consisting of the replacement of damaged tissue by newly formed tissue. This process proceeds through four successive phases: hemostasis; inflammation; proliferation, also known as replication and biosynthesis; and the remodeling phase [3,4,5]. The penultimate of these, the proliferative phase, includes three stages, i.e., biosynthesis of ECM components, epithelialization, and angiogenesis [4]. The interaction of cells with ECM components is regulated by biochemical mediators; numerous cytokines and growth factors, such as arachidonic acid derivatives (prostaglandins and leukotrienes); interleukins; interferons; TNF-a; PDGF; HGF; FGF; TGF; or EGF [6,7]. The first of the mentioned mediators participate in shaping the inflammatory response, and the next ones, i.e., growth factors, participate in controlling the proliferation, differentiation, and metabolism of cells involved in the healing process. The latter mediators also participate in the regulation of inflammatory processes; play a chemotactic role for neutrophils, monocytes/macrophages, fibroblasts, and epithelial cells (keratinocytes); and additionally stimulate angiogenesis and ECM formation [7]. The role of ECM components is related to the functional aspect of healing processes, as the above-mentioned components also perform signal transmission functions in this dynamic, interactive sequence of biological reactions [8,9]. Among the above-mentioned ECM components, the most important one—actively participating in all phases of healing—is hyaluronic acid, which performs both structural–biomechanical and regulatory functions in the discussed process [10,11,12]. In the early phase of healing—the phase of hemostasis—high-molecular-weight HA accumulates in the wound bed, and its molecules bind with fibrin and fibronectin, forming the so-called temporary matrix, facilitating the recruitment of inflammatory cells (neutrophils, macrophages, and lymphocytes) and fibroblasts [11,13,14]. With the onset of the next phase of healing—the inflammation phase—the next functions of hyaluronan, which this GAG performs in the course of the described process, become visible. GAG, in the form of fragments of low or intermediate molecular weight, activates the inflammatory phase, increasing the biosynthesis of proinflammatory cytokines, TNF-a, IL-1b, or IL-8, as a result of interaction with the CD44 receptor and the TOLL-like receptor (TLR4) [10,11]. Vascular endothelial cells stimulated by the action of TNF-a and IL-1b synthesize HA, which facilitates the adhesion of lymphocytes activated by the action of cytokines, expressing the CD44 receptor [10]. Hyaluronic acid can act as a moderator of the inflammatory phase as a result of free radical capture and interactions with hyaladherin—TSG-6 and serum protease inhibitor-LaL [10,15]. Interactions of hyaluronan with the TSG-6/IaI complex contribute to the reduction of the inflammatory response and then to the stabilization of granulation tissue, which is formed in the next phase of the healing process, i.e., the proliferative phase. In the discussed healing stage, HA stimulates not only the “tearing off” of fibroblasts from the matrix but also directly stimulates mitosis [10]. The matrix of early granulation tissue (up to the third day after damage) contains large amounts of hyaluronic acid and fibronectin. HA molecules, characterized by the ability to strongly swell, create a mesh that facilitates the penetration of the wound site by incoming cells [16]. Cell migration in the provisional matrix filling the damage site is stimulated by hyaluronan, as a result of the interaction of GAG with hyaladherins—CD44 and RHAMM [10]. Glycan affects the biosynthesis of collagens in the matrix, filling the damage site [17]. It has been shown that native hyaluronan, in a high-molecular-weight form, stimulates the expression of type III collagen during fetal healing and, in adults, in the early stages of tissue damage repair [15]. On the other hand, low-molecular-weight fragments of hyaluronic acid, formed, e.g., from 12 disaccharide units, enhance the expression of type I collagen—the dominant collagen in adult skin, the accumulation of which is intensified during scarring or fibrosis processes [15]. During the next stage of the proliferative phase, epithelialization and hyaluronan, through interaction with CD44 and RHAMM receptors, stimulates the migration and proliferation of keratinocytes. These phenomena are crucial both in the formation of the “correct” epidermis and in its “repair” [10,17]. The HA content in the healing wound bed changes as the repair processes progress [18,19]. This GAG is the dominant glycan of granulation tissue, and then in the subsequent stages of healing its content gradually decreases, reaching a plateau in the final phase of the discussed process [18,19].

Postoperative wound infection is the most common complication after median sternotomy. Many factors contribute to the risk, and treatment usually takes a long time and is often a challenge for the entire team of specialists [21]. The actions of medical personnel may be crucial, but it is also important to pay attention to the lifestyle, environmental conditions, age, or even gender of the patient.

### 4.1. Wound Classification

The classification of risk factors is divided into preoperative, intraoperative, and postoperative (Table 1).

### 4.2. Risk Factors for Wound Infection

*Preoperative*

Obesity (BMI > 30) is considered to be the main preoperative risk factor depending on the patient. Adipocyte hypertrophy, which increases the secretion of adipokines, increases the permeability of the vascular endothelium, allowing immune system cells to infiltrate into adipose tissue. Adipokines, together with de novo secreted cytokines, enhance the pathological immune response in tissues, closing the cycle leading to chronic inflammation [22]. There are many reports indicating that the patient’s gender influences the type of wound infection. Women have a higher risk of surgical site wound infection (SSWI), but men have a higher risk of deep sternal wound infection (DSWI) [23]. This is most likely due to the difference in chest circumference, which is smaller in women. However, in the case of female patients with significant breast size, an increased frequency of DSWI is noted, which may result from excessive tissue tension, promoting local ischemia and impaired healing processes [21]. Patient age is a significant risk factor (especially in patients over 70 years of age), mainly due to poor tissue regeneration and multimorbidity [22,23]. The literature indicates that smoking is a serious factor inducing wound infection. Sá MPBO et al. in a prospective analysis conducted on a group of 1500 patients qualified for coronary artery bypass grafting (CABG) showed a 2.1 times higher risk of DSWI (*p*-value 0.011; OR 2.67 (1.22–5.83)) compared to a group of non-smokers [24]. Chronic cough, resulting from smoking, may cause excessive tension and damage to the wires connecting the sternum, increasing the possibility of the separation of the sternum and bacterial infection of the wound and local necrosis [22]. Diabetic patients should have their blood glucose levels monitored preoperatively. Hyperglycemia is a condition that promotes immune system disorders and also affects wound healing. It has been shown that preoperative glucose levels of ≥200 mg/dL (≥11.1 mmol/L) increased the likelihood of DSWI by 10 times [21]. Long hospital stays in the preoperative period appear to be significant risk factors for SWI and DSWI. Storey A et al., in a prospective analysis, showed that in reports with shorter hospitalization, DSWI occurred less frequently compared to patients with longer hospitalizations. This condition may be caused by long exposure to antibiotic-resistant bacteria occurring in hospitals. Similar to resternotomy, the risk of postoperative infections is associated with emergency procedures, probably due to the short preparation time for the procedure and the patient’s hemodynamic instability. The risk of DSWI in preoperative hospital stays was presented in (Table 2) [25].

*Perioperative*

Perioperative management may be a factor affecting the frequency of the occurrence of sternal wound infection (SWI). Reports mainly indicate prolonged surgery time as a factor increasing the risk of infection. Prolonged opening of the chest leads to tissue drying and increased exposure to infectious agents [21,23,26]. Morrell Scott N et al. report that the risk occurs when the surgery lasts longer than 5 h, while Sajja LR. extends this time to 7 h [5,8]. Appropriate decontamination and disinfection of the surgical site should be performed carefully. Inadequate skin preparation will become a serious risk factor [27].

*Postoperative*

Resternotomy increases the incidence of DSWI by 6–9 times. Surgical damage inducing tissue ischemia disrupts wound healing processes. At the same time, prolonged exposure of the mediastinum to the external environment increases the risk of wound contamination and infection [21]. Medical procedures after median sternotomy, such as blood transfusion or ventilator use for more than 48 h, have been identified as additional risk factors. There is an ongoing discussion about the amount of blood units transfused to patients with preoperative or postoperative anemia that significantly increases the risk of DSWI. These values range from 2 to 4 blood units (RBC) [21,23].

#### Infectious Factors Disturbing the Healing Process

The identification of bacterial strains found in the wound is crucial for the introduction of targeted antibiotic therapy. In both SWI and DSWI, the most frequently isolated bacteria were Gram-positive, of which 60% are Staphylococcus aureus and Staphylococcus epidermidis, while 5–22% were Gram-negative rods Pseudomonas aeruginosa and *Acinetobacter baumani*. Fungi were sporadically an infectious factor. The remaining 25% of cases are multipathogenic infections [22]. The main habitats of pathogens are the patient’s skin, surgical instruments, medical equipment, the operating room environment, and objects brought during surgery [27]. It is worth emphasizing that the time from Staphylococcus aureus infection to the occurrence of symptoms may last from several weeks to months after the surgery [28].

### 4.3. Complications

The consequences of wound infection are complications that increase the significant deterioration of health or even death. In addition, they prolong the patient’s stay in the hospital and cause additional mental strain.

#### 4.3.1. Surgical Site Infection with Wound Dehiscence

The first symptoms shown by physical examination include elevated temperature (>38 °C), redness, and swelling of the skin around the wound, purulent discharge, chest pain, and sternal instability [22,29,30]. Differentiating SSWI from DSWI at an early stage is a diagnostic challenge. Imaging tests such as X-ray or CT scan are recommended for classification. An X-ray may show enlarged mediastinum or gas bubbles accumulating between the edges of the sternum. However, due to better image quality of soft tissues, CT scan is recommended [21,30]. Obtaining a wound culture is helpful in diagnosis and subsequent treatment planning. It is recommended to collect them from at least two wound sites for more accurate results [22]. SSWI is defined as a soft tissue infection without bone involvement. The term DSWI refers to infection involving the sternum, ribs, and mediastinum [21].

#### 4.3.2. Mediastinitis

Mediastinitis is one of the most serious complications associated with a high risk of mortality. The estimated incidence of mediastinitis is between 0.5 and 5% [31]. According to the Centers for Disease Control and Prevention (CDC) and the National Healthcare Safety Network (NHSN) 2024 guidelines, the diagnosis of mediastinitis should be based on at least one of the following criteria [32]:

1. An infectious agent should be identified in a microbiological examination of tissue or fluid collected from the mediastinum in a bacteriological culture or other diagnostic microbiological procedure that does not require culture to identify the infectious agent and/or implementation of appropriate therapeutic procedures.
2. The patient shows features of mediastinitis in a histopathological examination or physical examination based on chest observation.
3. The patient has one of the following symptoms: fever (>38.0 °C) and mediastinal pain or sternal instability without another cause.

In addition, the above symptoms are accompanied by one of the following:

- Purulent discharge from the mediastinum;
- Widened mediastinum on imaging.

4. For patients ≤1 year of age, one of the following symptoms is met: fever (>38.0 °C), hypothermia (<36.0 °C) and dyspnea, bradycardia, or sternal instability without other detectable cause.

In addition, the above symptoms are accompanied by one of the following:

- Purulent discharge from the mediastinum;
- Widening of the mediastinum silhouette in the imaging study.

In addition, mediastinitis after cardiac surgery is often accompanied by inflammation of the bone tissue of the sternum and bone marrow, which worsens the prognosis. The risk of mediastinitis after a previous cardiac surgery is increased by the following factors [23]:

- Age > 68 years;
- Chronic respiratory diseases/past respiratory diseases;
- Resternotomy 7 days after the previous surgery;
- Transfusion of blood products in the postoperative period;
- Intubation maintained >24 h after the surgery;
- Incidents of bleeding into the mediastinum and intrastinal hematomas [26].

There is an ongoing discussion on the risk of mediastinitis in correlation with a BIMA bypass. Numerous literature reports show significant discrepancies; however, it is assumed that this does not have a significant impact on the risk of developing inflammation in the postoperative period [33]. The main infectious factors responsible for the development of mediastinitis include Staphylococcus aureus occurring in 28–58.1% of cases of inflammation and *Acinetobacter* spp. responsible for approximately 20% of incidents [31]. Moreover, it has been shown that immunosuppressive treatment or alpha-blockers together with steroids is one of the factors increasing the risk of mediastinitis [23].

## 5. Prevention of Infections and Wound Healing Disorders After Sternotomy

Section 4.2 discusses factors that increase the risk of SWI. Infection prevention should focus on their elimination.

### 5.1. Asepsis and Antiseptics Before, During, and After Surgery

#### Identification of *S. aureus* Carriers and Patient Bathing

*S. aureus* carriers are three times more likely to develop surgical site infections than healthy individuals. Therefore, it is recommended to take a bacteriological nasal swab or order a test using polymerase chain reaction (PCR) as part of screening tests, no earlier than 12 h before surgery. Scientific reports have shown that the use of mupirocin (2% mupirocin ointment (Bactroban; GlaxoSmithKline, Research Triangle Park, NC) in carriers significantly reduced the risk of surgical site infection. Furthermore, its administration has not been shown to have any negative effect on non-carriers of *S. aureus* [34]. The aim of the maximum eradication of pathogens residing on the skin of patients undergoing planned surgeries is general body asepsis in the form of bathing with chlorhexidine agents [26].

### 5.2. Preoperative, Intraoperative, and Postoperative Factors

- Preoperative nutritional support

Patient malnutrition is associated with anemia and hypoproteinemia. Reduced erythrocyte count leads to tissue hypoxia and therefore to cellular hypoxia. There is also a decrease in the number of inflammatory cells along with immune cells, tissue edema, obstruction of granulation tissue, and collagen tissue synthesis. All together give a picture of slowed wound healing [22]. Morrell Scott N et al. in a retrospective analysis showed a significant relationship between patient malnutrition and increased SWI-related deaths [23]. Currently, there are no clear reports on whether feeding patients before a median sternotomy procedure reduces the risk of SWI and DSWI, while studies covering abdominal procedures have shown a significant reduction in the incidence of postoperative wound infection compared to patients (25.6% vs. 50.6%) [34].

- Blood glucose control (pre- and intraoperatively).

The analyzed reports have shown that blood glucose control is an effective prophylaxis in SWI and DSWI. Elevated blood glucose levels affect the immune system, which in turn affects the rate of wound healing. Hyperglycemia is associated with increased mortality, increased DSWI, and a prolonged hospital stay. A decrease in the number of SWI cases has been shown in patients with preoperative glucose levels of 100–150 mg/dL compared to patients with results of 250–300 mg/dL (1.3% vs. 6.7%). Appropriate insulin therapy in the preoperative period reduces the risk of DSWI by 63% [21,26].

- Quitting smoking.

Smoking correlates with an increased risk of mediastinal and sternal infections after CABG. It is recommended to stop smoking at least 30 days before the planned procedure [34]. At the same time, it has been shown that a complete reduction in smoking in the postoperative period is an important strategy to reduce the risk of DSWI [26].

- Controlled weight loss by the patient.

DSWI occurs in about 0.8% to 1.5% of patients with BMI < 30, and this value increases to 8% in obese patients [35]. Weight loss (BMI < 30 kg/m^2^) effectively reduces the frequency of SSWI and DSWI [26]

- Avoiding BIMA harvesting in patients at SWI risk.

Coronary artery bypass grafting with prior BIMA collection is associated with an increased probability of DSWI by 62% compared to LIMA collection. This is due to reduced blood supply to the sternum and adjacent structures. Elderly patients and those with diabetes are particularly at risk [29]. Obese people (BMI > 40) are at a significantly higher risk of impaired healing processes after BIMA collection due to the increased amount of tissue in the sternum. Therefore, the use of LIMA is recommended [26].

### 5.3. Closure Technique

In closing the integuments of the chest after a cardiac surgery, we can divide them into three main stages: hemostasis, an approximation of the edges of the sternum, and their anastomosis and closure of the skin integuments. Hemostasis as the first stage aims to stop bleeding into the mediastinum from the surrounding soft tissues and the periosteum and marrow cavity of the sternum. It is most often performed using electrocoagulation as well as bone wax (BW). Proper management should focus on reducing the frequency of electrocoagulation to the necessary minimum, thereby reducing further tissue damage [21]. Bone wax rubbed into the marrow cavity of the sternum allows for efficient and reliable hemostasis of the surgical site. The use of this hemostat has been a subject of discussion and controversy in the surgical community for many years. Özdemir AC et al. in a prospective analysis conducted on two groups of patients in whom wax was used or not used for hemostasis did not show statistical significance in relation to the later risk of complications in the postoperative period (*p* > 0.05). Moreover, no significant benefits were shown between the use of bone wax and the drainage volume in the 24 h observation after the procedure [36]. An alternative to bone wax is a new soluble polymer wax (WW). It is composed of hydrophilic alkylene oxide copolymers. This compound is absorbed from the sternum surface within 48 h and is excreted from the body in 90% by the kidneys. Vestergaard RF et al., in an analysis using CT imaging, showed a significant difference in the bone healing process, assessed semi-quantitatively on a scale of 0–10, (Table 3).

In addition, a significant improvement in physical fitness was demonstrated in patients who underwent WSW compared to BW within 1 and 3 months after the procedure. Six months after the surgical procedure, the WSW group was characterized by significantly better overall physical and mental health compared to BW [37].

### 5.4. Approximation and Fixation of the Sternal Edges

The approximation and fixation of the sternal edges is most often performed using steel wires. There are numerous techniques for placing a steel suture. The most commonly used are single, double, or type “8” wire closures. Shafi AMA et al., in a meta-analysis, showed the influence of the sternal wiring technique on the subsequent healing processes and the risk of complications. In the case of double wires, significantly better sternal stability was achieved. Destabilization occurred only in 2–5% of cases compared to single wire closures, where these values increased to 12–16%, *p* = 0.003, OR 0.25 (95% CI 0.10–0.63). Even better results in terms of sternal stability were obtained in the case of wire closure with the type “8” suture in relation to double wires, *p* = 0.04, OR 0.30 (95% CI, 0.09–0.96). Moreover, this technique, by relieving tension in the sternal area and reducing the likelihood of cutting bone tissue by the steel wire, was characterized by a significantly reduced frequency of sternal dehiscence and subsequent instability in comparison with single wires (0.06% vs. 0.66%; *p* < 0.0001) [38]. Malhotra A et al., in a prospective study comparing the technique of sternal approximation with the type “8” suture using polyester threads, noted a significantly higher frequency of complications in the form of postoperative wound infection or sternal dehiscence referring to the group of patients in whom a steel wire was used for closure with the same suture. [39] The closure of the skin layers is the last, very important stage of the surgical procedure. The tightness of the anastomoses is very important in creating a barrier between the mediastinum and surrounding tissues and the external environment. The most common technique for approximating the layers is a separate anastomosis of the subcutaneous tissues and the dermis edge to edge. In a prospective analysis, Steingrimsson S et al. compared the use of triclosan-coated and uncoated sutures. The 2-0 Vicryl Plus^®^ suture was used to close the subcutaneous tissues, and the 4-0 Monocryl Plus^®^ suture was used to approximate the skin edges intradermally. In one of the analyzed groups of patients, the sutures were additionally coated with Triclosan. No statistically significant effect of coated sutures on the reduction of the frequency of SWI incidents was demonstrated at 12.8% [95% confidence interval (CI) 8.7–18.5%] compared to uncoated sutures at 11.2% (95% CI 7.4–16.7%), (*p* = 0.640). Similar correlations were obtained for DSWI at 1.7% (95% CI 0.6–4.8%) and 2.2% (95% CI 0.8–5.6%), (*p* = 1.000) [40].

### 5.5. Prophylactic Antibiotic Therapy

Currently, regardless of the preferred guidelines (STS/AATS/EACTS), prophylactic antibiotic therapy is the recommended procedure for preventing surgical site infections. In comparison with the placebo, prophylactic antibiotic therapy reduces the risk of infection by an average of 1/5 [21,30]. In order to implement appropriate and safe prophylactic antibiotic therapy for the patient, it is necessary to determine the risk of MRSA skin colonization and exclude allergies to antibiotics from the penicillin and beta-lactam groups. Prophylactic antibiotic therapy for cardiac surgery patients is as follows:

- For the low-risk group of MRSA infection, the standard procedure is to administer an antibiotic from the beta-lactam group. In the case of choosing a first-generation cephalosporin (cefazolin), it is recommended to administer it within 60 min of skin incision.
- For the high-risk group of MRSA infection, there are three treatment regimens:
  ○ A beta-lactam antibiotic (recommended 1st or 2nd generation cephalosporin) with a glycopeptide (e.g., vancomycin);
  ○ Vancomycin with an antibiotic with a killing effect against Gram-negative strains (e.g., aminoglycoside).
- For the group of patients allergic to antibiotics from the penicillin/beta-lactam group, the administration of vancomycin is proposed together with an antibiotic with a killing effect against Gram-negative strains (e.g., aminoglycoside).

It should be emphasized that in the case of using cefazolin or cefuroxime in the event of a prolonged procedure (>4 h) or prolonged/excessive bleeding, it is necessary to re-administer the drug. This is due to the half-life of the above preparations and/or a decrease in their concentration in the blood during its loss.

The intraoperative use of vancomycin/gentamicin to cover the edges of the sternal incision is associated with a significant risk reduction from 5.8% to 2.0%. Kowalewski et al., in a 2015 meta-analysis, showed a 40% decrease in the frequency of post-sternotomy wound infections as a result of using a gentamicin–collagen sponge [21]. There are ongoing discussions regarding the appropriate duration of prophylactic antibiotic therapy to prevent surgical site infection. According to current guidelines, antibiotic therapy in the postoperative period is recommended for at least 24 h but no longer than 48 h. Extending the procedure >48 h does not reduce the risk of infection while increasing the likelihood of adverse events. Additionally, it is possible to induce drug-resistant strains or increase the likelihood of *Clostridium difficile* infection [21,30,34].

### 5.6. Postoperative Support

#### 5.6.1. Protection of the Chest with a Stabilizing Vest

Gorlitzer M et al., in a prospective analysis on a group of 2539 patients, showed a 54% reduction in the frequency of DSWI in patients using postoperative support in the form of a stabilizing vest. At the same time, no such relationship was observed in relation to SSWI. Moreover, the use of the above support did not affect the length of hospitalization [2].

#### 5.6.2. Postoperative Bathing of Patients

There is currently an ongoing discussion on the recommendations for bathing after median sternotomy in the postoperative period. A statistically significant reduction in the risk of SWI was proven in the group of patients using baths in the period of 48–72 h after the procedure. In the group of patients taking baths, the frequency of SWI was clearly lower compared to the control group (7.7% vs. 32%; *p* = 0.038). Moreover, patients reported better well-being and reduced pain on the VAS scale (1.54  ±  1.42 vs. 7.04  ±  1.24; *p* = 0.001) [41]. All risk factors categorized as modifiable and non-modifiable are presented in Table 4.

## 6. Treatment of Surgical Site Infections

There are currently no dedicated guidelines for the management of wound infection in patients after sternotomy. The choice of treatment method depends on the degree of tissue involvement in the postoperative wound. Treatment should focus on symptom control and the elimination of infectious processes, surgical debridement with adequate drainage, and finally the reconstruction and closure of the wound [22].

- Control and elimination of infectious processes.

The empiric treatment of wound infections should be based on the introduction of piperacillin/tazobactam or carbapenems. In cases of suspected or diagnosed MRSA infection, antibiotic therapy should be extended with vancomycin, daptomycin, or teicoplanin to eradicate the methicillin-resistant strain [21]. Moreover, linezolid has been shown to be an excellent antibiotic in infections with Gram-positive bacteria, leading to DSWI with osteomyelitis [22]. In the case of identification of infectious factors in a microbiological test, antibiotic therapy targeted to the pathogen is recommended [21].

- Surgical debridement of the wound and drainage.

The surgical debridement of the wound should be implemented in every situation when the infectious process extends to the sternum, regardless of its stability [35]. It consists of removing necrotic tissues in order to enable proliferation and granulation, leading to improved tissue blood supply and inducing wound healing processes. Previously used wound irrigation with povidone solution in the case of DSWI with concomitant mediastinitis is currently not recommended. The absorption of iodine solution by tissues during this process promotes iodine accumulation. This can lead to renal failure, electrolyte imbalance, changes in the thyroid hormone system, and also psychological changes. Currently, antibiotic solutions are recommended for wound irrigation [34]. Early surgical intervention in DSWI is associated with a significantly shorter hospitalization period compared to patients whose intervention was delayed for more than 7 days from the diagnosis of DSWI [21]. Wound drainage is an extremely important element of therapeutic intervention. The most commonly used technique is a negative pressure dressing (NPWT). A dedicated polyurethane foam placed in the wound, sealed with a foil with a connected suction system, allows for continuous removal of fluids and tissue from the wound. This helps improve blood supply to the granulation tissue. Additionally, the sucked-in foam stiffens and stabilizes the sternum [21]. Randomized clinical trials have shown the advantage of NPWT both when used alone and in combination with previous surgical debridement of the wound. Patients undergoing NPWT therapy had shorter hospitalizations, and negative wound culture results were obtained more quickly. In addition, the use of the therapy shortened the time between the surgical debridement of the wound and its final closure. Mortality in the group of patients with mediastinitis who received NPWT was 6% compared to 25% in the control group [34].

- Reconstruction and closure of the wound.

After surgical debridement of the wound, a decision should be made about its closure or reconstruction. In the absence of infectious process markers and the possibility of sufficient approximation of the edges of the sternum to obtain satisfactory stabilization, direct wound closure is possible. In the cases of significant tissue loss of the sternum and muscles, it is necessary to use muscle grafts for reconstruction [21]. The muscles most often used to reconstruction are as follows: pectoralis major, rectus abdominis, and latissimus dorsi. In addition, fragments of the greater omental sac are used. Due to the location and the possibility of avoiding a new surgical incision constituting an additional site of infection, it is recommended to use the pectoralis major muscle if possible [27,42].

The pectoralis major muscle (PM) was the first muscle used as a tissue graft in patients with DSWI. The possibility of unilateral or bilateral harvesting and the lack of the need to perform another incision make it possible to consider this muscle as the default muscle for use in the event of the need to supply a wound after sternotomy with a tissue graft. Bilateral muscle excision compared to unilateral muscle excision is characterized by better wound stabilization and the reduction of the free space between the muscle and the surrounding tissues. PM is recommended for supplying defects of the upper 2/3 of the sternum. In addition, the excision of the graft together with the perforator, which is the internal thoracic artery, promotes a better healing process compared to the excision of the fragment not supplied with a vascular perforator.

The rectus abdominis muscle (RA), supplied by the upper and lower epigastric arteries, is a graft in the case of tissue reconstruction of the lower 1/3 of the sternum. It is possible to use just the muscle or a skin-tissue graft. It is recommended to use RA in the case of previous coronary artery bypass grafting using BIMA. Additionally, in the case of the need to provide more extensive DSWI, the PA muscle graft is anastomosed with RA, which significantly improves the prognosis of patients. Harvesting RA is associated with a certain risk of abdominal hernia. This can be reduced by leaving the fascia and closing the wound in two layers.

The latissimus dorsi (LD) is an alternative muscle graft in the case of BIMA bypass and poorly developed perforators. Harvesting does not disrupt blood flow in the sternum and surrounding tissues and reduces the risk of dysfunction of the shoulder girdle and respiratory muscles compared to the PA graft. The LD flap is used to supply extensive tissue defects surrounding the sternum during DSWI. The main disadvantage is the increased risk of complications in the area of the harvest site and the need for intraoperative change of the patient’s position to obtain the LD flap.

The greater omental sac is used to cover tissue defects when it is impossible to use muscle flaps from the PM and RA. Supply through the gastroepiploic arteries provides exceptionally good perfusion that promotes the penetration of nutrients and used pharmaceuticals in the surrounding tissues. Additionally, in the case of the need for a simultaneous skin graft, it is possible to place it directly on the tissue cover from the greater omental sac without waiting for the formation of granulation tissue. It is sometimes used together with bilateral PA muscle flap harvesting to reconstruct the wound in the lower pole of the sternum and reduce the overall postoperative mortality. The disadvantage of this method is the need for a diaphragm incision [27].

## 7. Discussion

Postoperative wound infection is one of the significant threats to patients after previous cardiac surgery. The mediastinum, as a closed cavity, is strictly isolated. Median sternotomy opens it, exposing the cavity to pathogens from the external environment. The prevention and treatment of infections requires a multidisciplinary approach and the implementation of new techniques that constantly improve the prognosis of patients. It is worth emphasizing that the risk of postoperative complications in the form of surgical site infection is influenced by numerous factors, both in the preoperative, intraoperative, and postoperative periods. A correct diagnosis based on the first symptoms of infection is crucial for implementing appropriate treatment in the early stages. The above actions are intended to avoid the most serious complication, with a high mortality rate in the form of mediastinitis. Due to the lack of guidelines, the treatment procedure is strictly dependent on the degree of tissue involvement and the clinical picture presented. It should focus on several steps, starting with attempts to use targeted antibiotic therapy, surgical intervention to cleanse the wound with the establishment of drainage, or an attempt to approximate the wound edges. As a last resort, reconstructive surgery with tissue flap transplants is used, especially in the case of significant bone tissue loss in the sternum. The prevention of wound infection includes effective aseptic and antiseptic techniques, avoiding or reducing risk factors, and choosing the appropriate wound closure technique for a given patient. The use of prophylactic antibiotic therapy and appropriate postoperative support can significantly reduce the risk of wound healing disorders. New technologies, such as silver nanocrystal-coated dressings or the use of platelet-rich plasma in the wound healing process, provide hope for a better future.

## 8. Conclusions

Complications of wound healing after sternotomy are currently a significant therapeutic problem in cardiac surgery patients.

Despite significant progress in the prevention and treatment of surgical site infections in recent years, it is necessary to continue clinical trials to find better, alternative techniques and to control the safety of currently used ones. This creates new challenges for both surgical specialists and nursing and rehabilitation staff who have direct contact with patients.

The above analysis aims to synthesize and summarize modifiable and non-modifiable SSWI and DSWI risk factors in order to simplify the work of clinicians.

Impact on modifiable risk factors and their potential elimination will improve patient prognosis and reduce the risk of infection. In addition, knowledge of non-modifiable factors will influence the implementation of prevention at an early stage of treatment to prevent complications.

## Figures and Tables

**Table 1:** Surgical site wound infection (SSWI) and deep sternal wound infection (DSWI) [21].

Classification of Post-Sternotomy Wound Infections		
	Type	Degree of tissue involvement
SSWI	1	Skin and subcutaneous tissue
DSWI	2a	Retrosternal tissues without involving bone
	2b	Retrosternal tissues
	2c	Retrosternal tissues, involving bone
	2d	Frank osteitis

**Table 2:** Deep sternal wound infection (DSWI) [25].

Risk of DSWI Occurrence in Relation to the Postoperative Hospital Length of Stay	
Preoperative hospital stay [days]	OR
2	OR 1.4, 95% CI 1.06–2.01
3	OR 1.9, 95% CI 1.2–3.8/OR 2.1, 95% CI 1.1–4.1
5	OR 2, 95% CI 1.2–3.5
7	OR 1.6, 95% CI 1.1–2.5

**Table 3:** Computer tomography (CT) [37].

Semi-Quantitative Assessment of the Degree [0–10] of Bone Tissue Healing in CT Scan		
3 months after surgery		
	Semi-quantitative scale [0–10]	*p*-value
Bone wax	0.6 ± 0.9	*p* < 0.05
Polymer soluble wax	2.6 ± 1.7	
Control group	2.7 ± 1.5	
6 months after surgery		
Bone wax	3.3 ± 2.5	*p* < 0.001
Polymer soluble wax	6.5 ± 0.4	
Control group	6.9 ± 2.4	

**Table 4:** Risk factors categorized as modifiable and non-modifiable.

Hospitalization Stage	Modifiable	Non-Modifiable
Preoperative	Obesity	Sex (Female)
	Nutritional support	
	Smoking	
	High blood glucose level (Diabetic)	Age
	Prophylactic antibiotic therapy	
Perioperative	Adequate skin and wound asepsis, antiseptic	Blood transfusion
	Surgery time	
	Avoid BIMA harvest	Resternotomy
	Closure technique	
	Sternal edges fixation	
Postoperative	Long hospitals stays	
	Ventilation > 48 h	
	Chest stabilizing vest	
	Postoperative bathing	

## Data Availability

On request of those interested.

## Abbreviations

SSWI	Surgical site wound infection
SWI	Sternal wound infection
DSWI	Deep sternal wound infection
CABG	Coronary artery bypass grafting
RBC	Red blood cells, erythrocytes
BMI	Body mass (weight) to height ratio
CDC	The Centers for Disease Control and Prevention
NHSN	The National Healthcare Safety Network
PCR	Polymerase chain reaction
BIMA	Technique using both internal mammary arteries
LIMA	Left internal thoracic artery
BW	Bone wax
CT	Imaging
STS/AATS/EACTS	Expert consensus on post-cardiotomy extracorporeal life support in adult patients
MRSA	Methicillin-resistant staphylococcus aureus
PM	Pectoralis major muscle
RA	Rectus abdominis muscle
LD	Latissimus dorsi

## References

[1] Bochenek A., Reicher M. (1993). Human Anatomy.

[2] Gorlitzer M., Wagner F., Pfeiffer S., Folkmann S., Meinhart J., Fischlein T., Reichenspurner H., Grabenwoeger M. (2013). Prevention of sternal wound complications after sternotomy: Results of a large prospective randomized multicentre trial. Interact Cardiovasc. Thorac. Surg..

[3] Chin G.A., Diegelmann R.F., Schultz G.S., Falabella A.F., Kirsner R.S. (2005). Cellular and molecular regulation of wound healing. W: Wound Healing.

[4] Garner W., Nabavian R., Sood R., Aucher B.M. (2006). Normal wound healing. W: Reconstruction and Rehabilitation.

[5] Watson T. Soft Tissue Wound Healing Review. https://www.electrotherapy.org/tissue-repair.

[6] Hantash B.M., Zhao L., Knowles J.A., Lorenz H.P. (2008). Adult and fetal wound healing. Front. Biosci..

[7] Sommer C.V., Porth C.M. (2007). Inflammation, tissue repair, and fever. W: Essentials of Pathophysiology.

[8] Dylewski M.L., Yu Y.M., Molnar J. (2007). Protein and wound healing. W: Nutrition and Wound Healing.

[9] Li J., Kirsner R.S., Falabella A.F., Kirsner R.S. (2005). Extracellular matrix and wound healing. W: Wound Healing.

[10] Dechert T.A., Ducale A.E., Ward S.I., Yager D.R. (2006). Hyaluronan in human acute and chronic dermal wounds. Wound Repair Regen..

[11] Chen W.Y., Abatangelo G. (1999). Functions of hyaluronan in wound repair. Wound Repair Regen..

[12] Slevin M., Kumar S., Gaffney J. (2002). Angiogenic oligosaccharides of hyaluronan induce multiple signaling pathways affecting vascular endothelial cell mitogenic and wound healing responses. J. Biol. Chem..

[13] Pogrel M.A., Pham H.D., Guntenhöner M., Stem R. (1993). Profile of hyaluronidase activity distinguishes carbon dioxide laser from scalpel wound healing. Ann. Surg..

[14] Riessen R., Wight T.N., Pastore C., Henley C., Isner J.M. (1996). Distribution of hyaluronan during extracellular matrix remodeling in human restenotic arteries and balloon-injured rat carotid arteries. Circulation.

[15] David-Raoudi M., Tranchepain F., Deschrevel B., Vincent J.C., Bogdanowicz P., Boumediene K., Pujol J.P. (2008). Differential effects of hyaluronan and its fragments on fibroblasts: Relation to wound healing. Wound Repair Regen..

[16] Olczyk P., Komosińska-Vassev K., Winsz-Szczotka K., Kuźnik-Trocha K., Olczyk K. (2008). Hyaluronan: Structure, metabolism, functions, and role in wound healing. Postępy Hig. Med. Doświadczalnej.

[17] Price R.D., Berry M.G., Navsaria H.A. (2007). Hyaluronic acid: The scientific and clinical evidence. J. Plast. Reconstr. Aesthetic Surg..

[18] Bentley J.P. (1967). Rate of chondroitin sulfate formation in wound healing. Ann. Surg..

[19] Siméon A., Wegrowski Y., Bontemps Y., Maquart F.X. (2000). Expression of glycosaminoglycans and small proteoglycans in wounds: Modulation by the tripeptide-copper complex glycyl-L-histydyl-L-lysine-Cu^2+^. J. Investig. Dermatol..

[20] Tribble C., Merrill W., Derryberry S., Parrino G. (2021). The Median Sternotomy: The Unkindest Cut of All? Pearls, Pitfalls, Aphorisms, & Myths. Heart Surg. Forum.

[21] Phoon P.H.Y., Hwang N.C. (2020). Deep Sternal Wound Infection: Diagnosis, Treatment and Prevention. J. Cardiothorac. Vasc. Anesth..

[22] Song Y., Chu W., Sun J., Liu X., Zhu H., Yu H., Shen C. (2023). Review on risk factors, classification, and treatment of sternal wound infection. J. Cardiothorac. Surg..

[23] Morrell Scott N., Lotto R.R., Spencer E., Grant M.J., Penson P., Jones I.D. (2022). Risk factors for post sternotomy wound complications across the patient journey: A systematised review of the literature. Heart Lung.

[24] Sá M.P.B.O., Ferraz P.E., Soares A.F., Miranda R.G.A., Araújo M.L., Silva F.V., Lima R.C. (2017). Development and Validation of a Stratification Tool for Predicting Risk of Deep Sternal Wound Infection after Coronary Artery Bypass Grafting at a Brazilian Hospital. Braz. J. Cardiovasc. Surg..

[25] Storey A., MacDonald B., Rahman M.A. (2021). The association between preoperative length of hospital stay and deep sternal wound infection: A scoping review. Aust. Crit. Care.

[26] Sajja L.R. (2015). Strategies to reduce deep sternal wound infection after bilateral internal mammary artery grafting. Int. J. Surg..

[27] Hever P., Singh P., Eiben I., Eiben P., Nikkhah D. (2021). The management of deep sternal wound infection: Literature review and reconstructive algorithm. JPRAS Open.

[28] van Wingerden J.J., Maas M., Braam R.L., de Mol B.A. (2016). Diagnosing poststernotomy mediastinitis in the ED. Am. J. Emerg. Med..

[29] Gudbjartsson T., Jeppsson A., Sjögren J., Steingrimsson S., Geirsson A., Friberg O., Dunning J. (2016). Sternal wound infections following open heart surgery—A review. Scand. Cardiovasc. J..

[30] Abu-Omar Y., Kocher G.J., Bosco P., Barbero C., Waller D., Gudbjartsson T., Sousa-Uva M., Licht P.B., Dunning J., Schmid R.A. (2017). European Association for Cardio-Thoracic Surgery expert consensus statement on the prevention and management of mediastinitis. Eur. J. Cardiothorac. Surg..

[31] Yuan S.M. (2015). Sternal wound tuberculosis following cardiac operations: A review. Rev. Bras. De Cir. Cardiovasc..

[32] (2024). National Healthcare Safety Network (NHSN) Site; Chapter 17: CDC/NHSN Surveillance Definitions for Specific Types of Infections. https://www.cdc.gov/nhsn/psc/vae/index.html.

[33] Vitulli P., Frati G., Benedetto U. (2015). Bilateral internal mammary artery grafting in obese: Outcomes, concerns and controversies. Int. J. Surg..

[34] Lazar H.L., Salm T.V., Engelman R., Orgill D., Gordon S. (2016). Prevention and management of sternal wound infections. J. Thorac. Cardiovasc. Surg..

[35] Alebrahim K., Al-Ebrahim E. (2020). Prevention, Classification and Management Review of Deep Sternal Wound Infection. Heart Surg. Forum.

[36] Ozdemir A.C., Aykut K. (2014). Efficacy and safety of bone wax to reduce sternal bleeding following coronary bypass surgery. Ann. Thorac. Cardiovasc. Surg..

[37] Vestergaard R.F., Nielsen P.H., Terp K.A., Søballe K., Andersen G., Hasenkam J.M. (2014). Effect of hemostatic material on sternal healing after cardiac surgery. Ann. Thorac. Surg..

[38] Shafi A.M.A., Abuelgasim E., Abuelgasim B., Iddawela S., Harky A. (2021). Sternal closure with single compared with double or figure of 8 wires in obese patients following cardiac surgery: A systematic review and meta-analysis. J. Card. Surg..

[39] Malhotra A., Garg P., Bishnoi A.K., Pendro V., Sharma P., Upadhyay M., Gandhi S. (2014). Is steel wire closure of sternotomy better than polyester suture closure?. Asian Cardiovasc. Thorac. Ann..

[40] Steingrimsson S., Thimour-Bergström L., Roman-Emanuel C., Scherstén H., Friberg Ö., Gudbjartsson T., Jeppsson A. (2015). Triclosan-coated sutures and sternal wound infections: A prospective randomized clinical trial. Eur. J. Clin. Microbiol. Infect. Dis..

[41] Gök F., Demir Korkmaz F., Emrecan B. (2022). The effects of showering in 48–72 h after coronary artery bypass graft surgery through median sternotomy on wound infection, pain, comfort, and satisfaction: Randomized controlled trial. Eur. J. Cardiovasc. Nurs..

[42] Kaul P. (2017). Sternal reconstruction after post-sternotomy mediastinitis. J. Cardiothorac. Surg..

